# Steric effects and quantum interference in the inelastic scattering of NO(X) + Ar†
†Electronic supplementary information (ESI) available. See DOI: 10.1039/c4sc03842h



**DOI:** 10.1039/c4sc03842h

**Published:** 2015-02-03

**Authors:** B. Nichols, H. Chadwick, S. D. S. Gordon, C. J. Eyles, B. Hornung, M. Brouard, M. H. Alexander, F. J. Aoiz, A. Gijsbertsen, S. Stolte

**Affiliations:** a The Department of Chemistry , University of Oxford , The Physical and Theoretical Chemistry Laboratory , South Parks Road , Oxford , OX1 3QZ , United Kingdom . Email: mark.brouard@chem.ox.ac.uk; b Department of Chemistry and Biochemistry and Institute of Physical Science and Technology , University of Maryland , College Park , MD 20742 , USA . Email: mha@umd.edu; c Departamento de Química Física , Facultad de Química , Universidad Complutense , 28040 Madrid , Spain . Email: aoiz@quim.ucm.es; d Institute for Lasers, Life and Biophotonics , Vrije Universiteit , de Boelelaan 1083 , Amsterdam 1081 HV , The Netherlands; e Institute of Atomic and Molecular Physics , Jilin University , Changchun 130012 , China . Email: s.stolte@vu.nl; f Department of Physics and Astronomy , LaserLaB , Vrije Universiteit , de Boelelaan 1083 , Amsterdam 1081 HV , The Netherlands; g Laboratoire Francis Perrin , Bâtiment 522, DRECEM/SPAM/CEA Saclay , 91191 Gif sur Yvette , France

## Abstract

Rotationally inelastic collisions of NO(X) with Ar are investigated in unprecedented detail using state-to-state, crossed molecular beam experiments. The NO(X) molecules are selected in the *Ω* = 0.5, *j* = 0.5, *f* state and then oriented such that either the ‘N’ or ‘O’ end of the molecule is directed towards the incoming Ar atom. Velocity map ion imaging is then used to probe the scattered NO molecules in well-defined quantum states. We show that the fully quantum state-resolved differential steric asymmetry, which quantifies how the relative efficiency for scattering off the ‘O’ and the ‘N’ ends of the molecule varies with scattering angle, is strongly affected by quantum interference. Significant changes in both integral and differential cross sections are found depending on whether collisions occur with the N or O ends of the molecule. The results are well accounted for by rigorous quantum mechanical calculations, in contrast to both classical trajectory calculations and more simplistic models that provide, at best, an incomplete picture of the dynamics.

## Abstract 2

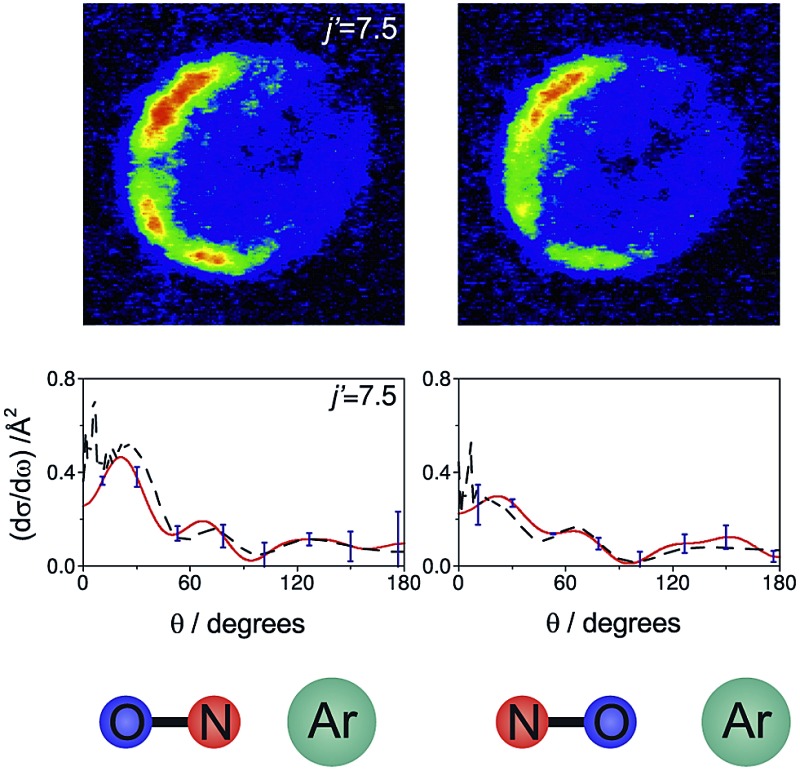
New measurements of the differential steric effect for NO + Ar inelastic scattering highlight the importance of quantum interference.

## Introduction

Much of chemical kinetics can be understood on the basis of Newtonian mechanics. Nuclei follow “quasi-classical” trajectories (QCT) guided by forces which are the gradients of potential energy surfaces. That said, in many systems, such as the diatom–atom system studied in this work, quantum interference occurs between collisions which follow different classical trajectories. Studying these interferences has formed the focus of many experimental and theoretical investigations.

Quantum scattering simulations of collisions of “near-homonuclear” (almost symmetrical) molecules such as CO with noble gasses provided an early prediction of interference.^1^ Cross sections for transitions with even changes in the rotational angular momentum (or, equivalently, conservation of the total parity of the rotational states) were significantly larger than those with odd changes in rotational state, *j* (and a change in the total parity). This alternation is a manifestation of quantum interference, and cannot be predicted by QCT calculations. In a semi-classical explanation, quantum phases are associated with each trajectory leading from a particular initial to a particular final state.^2,3^

In a typical experiment, the partner will collide with one or the other end of the molecule. Since neither end is selected, the observation will be subject to quantum interference. Just as in the textbook double-slit experiment, each trajectory accumulates a complex phase. These will interfere, constructively or destructively, in any experiment which monitors only the initial and final states of the collision partners.

Because of experimental accessibility, crossed molecular beam collisions of Ar with NO, a near-homonuclear molecule, provided the first laboratory confirmation of this effect.^4^ Subsequent experiments measured not only integral (ICS) but also differential (DCS) cross sections.^5–9^ DCSs are sensitive to a more specific type of quantum interference, between different trajectories which end up at the same laboratory scattering angle.

There is a third source of quantum interference: the outermost electron in NO occupies a doubly-degenerate π-type anti-bonding molecular orbital. Approach of a collision partner lifts this degeneracy, which results in two different potential energy surfaces which are both sampled, coherently, during the collision.^10^

A beautiful series of studies have probed these interferences in increasingly state-selective molecular-beam experiments. In NO the electronic degeneracy manifests itself in a splitting of each rotational level into closely-spaced Λ-doublets, of opposite parity, labeled *e* and *f*. Earlier experiments measured DCSs for an incoherent mixture of the *e* and *f* Λ-doublet initial states.^5–8,11^ More recently, use of a hexapole electric field or Stark decelerators have made possible similar experiments with NO selected in a defined-parity, single Λ-doublet level.^9,12,13^ In addition to measuring the angular distribution of the scattered products, or DCS, more complex experiments allow determination of the plane^8,11,14,15^ or sense^6,14^ of the rotation (or equivalently the alignment or orientation of the rotational angular momentum *j*′) as a function of scattering angle.

To elucidate the three dimensional steric properties of a collision, it is necessary to determine how the angular scattered product distribution changes with the orientation of the molecule relative to the direction of approach. To date no differential scattering experiments have been performed which answer the most chemical question about the interaction of a partner with NO: do collisions with the ‘N’-end lead to a greater or lesser degree of rotational excitation, than collisions with the ‘O’-end, as a function of scattering angle? This difference is the differential “steric asymmetry”.

Here, we use a static electric field to generate a coherent superposition of the two Λ-doublets of NO in its lowest rotational level. This allows us to control the orientation of the bond axis, defined by the vector ***r***, prior to collision,^3,16–19^ in other words, to select the ‘O’ or ‘N’ orientation of the molecule. Measuring the angular dependence of the scattering of NO so prepared will yield the so-called three vector ***k***–***r***–***k*′** correlation (where ***k*** and ***k*′** are the initial and final relative momenta), or oriented differential cross section. This will be the inelastic analogue of recent experiments on the Cl + CHD_3_ reaction, which measured the three-vector ***k***–***j***–***k*′** correlation in a reactive collision.^20^ Classically, the initial angular momentum, ***j***, is perpendicular to ***r***, so orientation or alignment of ***j*** also provides information on the direction of ***r***. In our experiment we can prepare molecular quantum coherences and observe how they are transformed by the collision.

This paper is laid out as follows: sections A and B provide details of the experimental method, including the orientation of the NO(X) molecule. The details of the theoretical methods employed in this study; quantum mechanical scattering calculations and quasi-classical trajectory calculations are provided in section C. Results sections D and E present the integral and differential steric asymmetry results for NO(X) + Ar scattering respectively. Conclusions then follow.

## Methods

### A Experimental methods

**Fig. 1:**
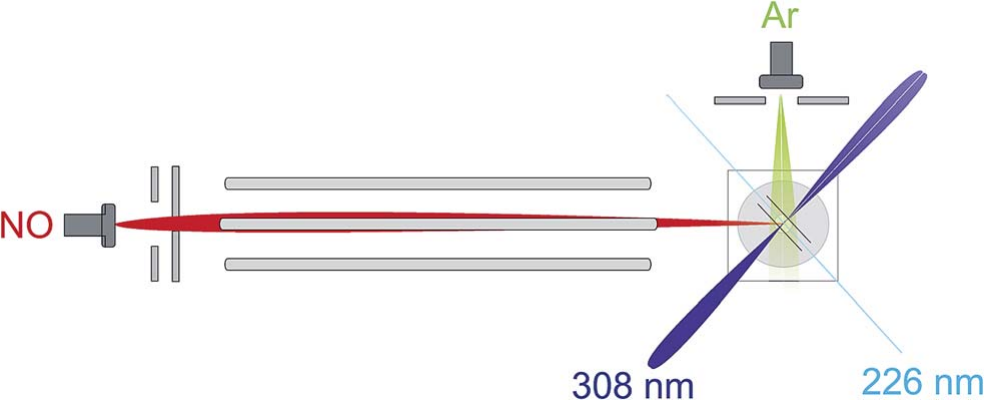
An overview of the experimental apparatus, as described in the main text.

An overview of the experiment is shown schematically in Fig. 1. We employ a crossed molecular beam apparatus, coupled with hexapole initial quantum state selection and (1 + 1′) resonantly enhanced multiphoton ionization (REMPI) velocity-mapped ion imaging final state detection.^9,12^ Both molecular beams are formed using pulsed general valves at a backing pressure of 3 bar. The primary beam contains NO seeded at 16% in Ar and is doubly skimmed before entering the hexapole and collimated on entrance to the scattering chamber. The secondary beam consists of pure Ar and is skimmed by a single skimmer approximately 8 cm from the scattering centre. Firing the secondary beam at half the frequency of the primary allows the unscattered NO background to be recorded and subtracted on a shot by shot basis.^12^ Simulations suggest that the beam conditions employed yield an approximately Gaussian collision energy distribution with a mean of 530 cm^–1^, and a full-width-at-half-maximum (FWHM) of 50 cm^–1^, as in previous studies.^12^

After the adiabatic expansion, the majority of the NO(X) molecules in the beam are in their rotational ground state, however the populations of the *e* and *f* Λ-doublet levels are near equal due to the small energy splitting between them (0.01180 cm^–1^). Initial state selection of the NO(X) is therefore achieved using a hexapole electric field, which exploits the Stark effect to select the low field seeking |*Ω* = 0.5, *j*′ = 0.5, *f* state and focusses it into the interaction region.^9,12^ Molecules in the high field seeking *e* Λ-doublet level are expelled from the hexapole electric field and higher rotational states are defocussed due to their weaker Stark effect.^9,12^

**Fig. 2:**
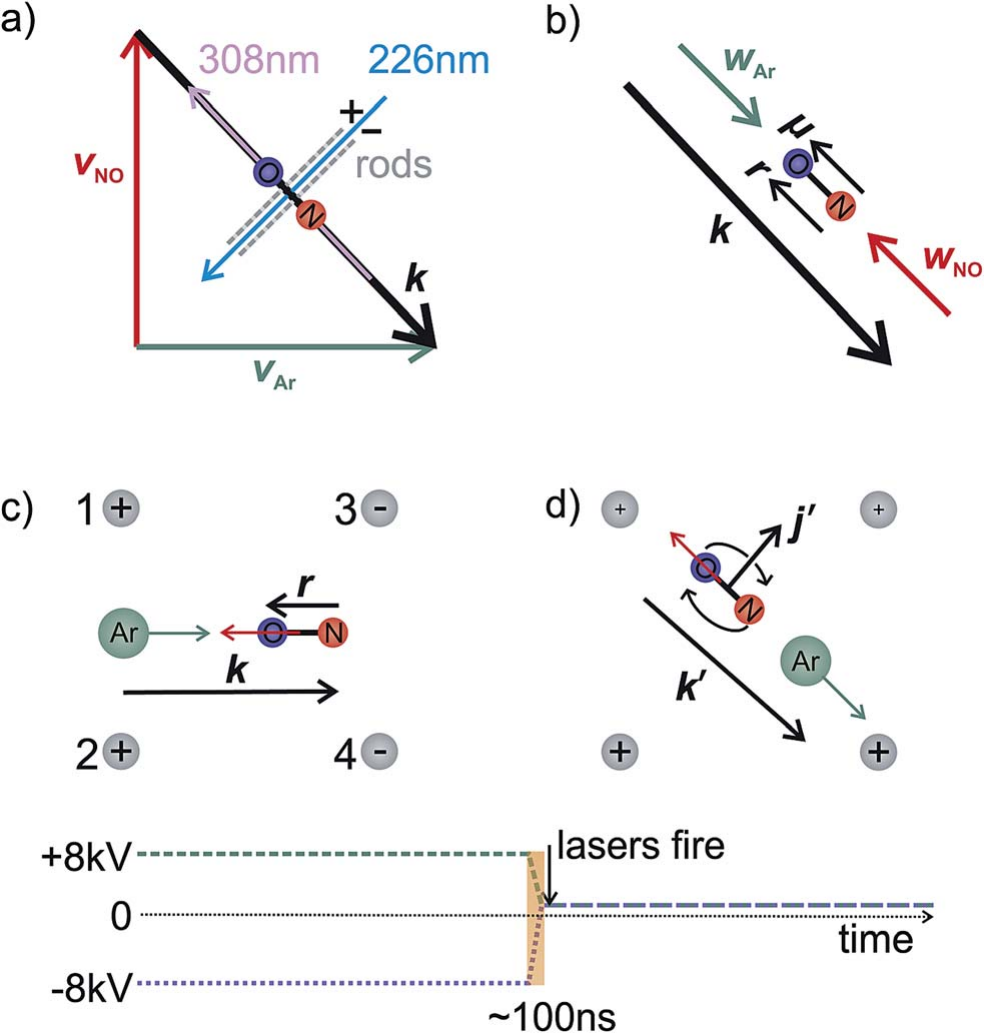
Panel (a) illustrates the interaction region, indicating the directions of the molecular and laser beams and orientation rods. Panel (b) shows the relative and centre-of-mass velocities and the orientation of the NO. An ‘O’ end collision is assumed. The lower panels (c and d) show a cross section through the orientation rods, illustrating the four vectors necessary to fully describe the motion before (panel c) and after (panel d) collision. Below panels (c) and (d), the voltage applied to rods 1 and 2 is shown by the green dashed line, whilst the blue dotted line indicates the voltage applied to rods 3 and 4. In panel (c), the electric field orients the bond axis of the molecule along *k* such that the ‘O’ end of the molecule is directed towards the Ar. After the collision, the voltage is switched to velocity mapping settings (approximately +1 kV), and the scattered NO molecules imaged onto the detector. *k*′ and *j*′ are the final relative velocity and rotational angular momentum vectors, respectively. Every 1000 shots the direction of the field is switched to allow alternate recording of both orientations.

In the interaction region the NO molecules are exposed to a static electric field, generated by a four-rod electrode. The rods lie perpendicular to the relative velocity, ***k*** (shown by the black arrow in panel (a) of Fig. 2). Depending on its direction, the field orients the bond axis, ***r***, of the NO molecules either parallel or antiparallel to ***k*** as described in the following section. A cross section through the rods and interaction region is shown in panels (c) and (d) of Fig. 2.

As shown in Fig. 2(c), a negative voltage (–8 kV) is applied to rods 3 and 4, and a positive voltage (+8 kV) to rods 1 and 2, resulting in an electric field of approximately 9.2 kV cm^–1^ that orients the ‘O’ end of the NO molecule towards the incoming Ar atom. (1 + 1′) resonant enhanced multiphoton ionization (REMPI) is then used to ionize selectively the scattered NO molecules. The probe laser is tuned to individual rotational lines of the NO(A ← X) transition at wavelengths around 226 nm. The electronically excited NO molecules are then ionized using 308 nm radiation from a XeCl excimer laser. This detection scheme allows observation of the quantum state resolved DCS, with the identity of the rotational branch determining the final Λ-doublet level probed. Scattered NO molecules arising from collisions populating the final *e* Λ-doublet level, as presented in sections D and E, are probed from an analysis of data recorded on the R_11_ and overlapping Q_21_ satellite branches. Velocity mapped^21^ ion imaging^22^ is then used to map the resulting ions onto a position sensitive detector. To achieve velocity mapping conditions, approximately 100 ns before the lasers are fired, the voltages applied to the rods are rapidly switched such that approximately +1 kV is applied to all four rods. The extraction field employed to velocity map the NO ions is insufficient to mix the NO(X) Λ-doublet levels and orient the NO. Ions are detected using a standard MCP/phosphor screen system, with the flashes on the phosphor screen recorded using a charge-coupled device (CCD) camera. Data are then transferred to a PC for subsequent averaging and data analysis. After 1000 laser shots the direction of the orienting field is then reversed to allow recording, alternately, ‘O’ end (NO–Ar) and ‘N’ end (ON–Ar) images.

Ion images are recorded with the probe laser polarization aligned both in the plane of the molecular beams (H) and perpendicular to it (V). The 308 nm excimer laser radiation was unpolarized. Both sets of images are then analysed and the DCSs extracted from each set averaged, as described in more detail in the ESI.†

### B Orientation of NO(X)

In the X ^2^Π_*Ω̄*_ electronic ground state of NO there are two spin–orbit manifolds, ^2^Π_1/2_ and ^2^Π_3/2_, the latter of which lies about 123 cm^–1^ higher in energy. In addition, each rotational level within the spin–orbit manifolds is split into two near degenerate Λ-doublet levels, distinguished by the symmetry index *ε*, which can take values of +1 (labelled *e*) and –1 (labelled *f*). The total parity of the NO(X) wavefunction is given by *p* = *ε*(–1)^*j*–1/2^.

The NO molecular wavefunction in the Hund's case (a) coupling scheme can be written as^23^ 
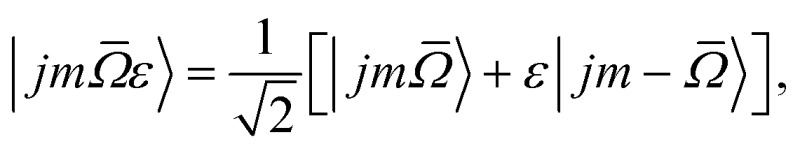
where *j* is the total angular momentum quantum number apart from nuclear spin, with projections *m* and *Ω* onto the space and molecule fixed axes respectively. Note that *Ω̄* is the absolute value of projection of the total electronic angular momentum along the internuclear axis.

In our experiments the initial state selection of the NO(X) molecule is achieved using a hexapole electric field which exploits the Stark effect to select only the |*Ω̄* = 0.5, *j*′ = 0.5, *ε* = –1, *f* state.^12^ Hexapole state selection thus focusses only the *f* Λ-doublet level into the interaction region, and the NO molecules are then exposed to a static electric field used to orient the bond axis. In a pure Hund's case (a) basis, which is reasonable for low NO rotational states, the NO molecular wavefunction in a static electric field can be written as a linear combination of the field free *e* and *f* states,^18,19,24,25^
1
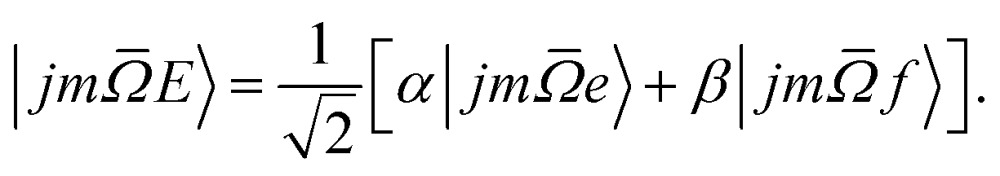



The relative signs of the mixing coefficients, *α* and *β*, are discussed further below. Their magnitudes are given in terms of the strength of the reduced electric field, *E*_red_, as2
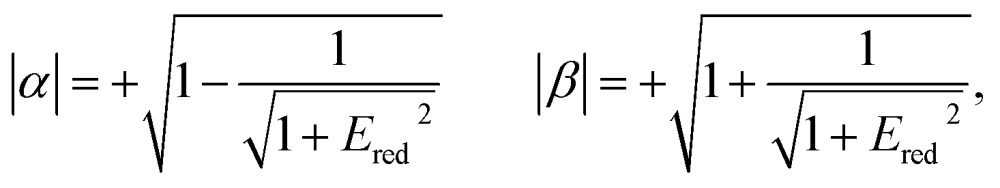
where3
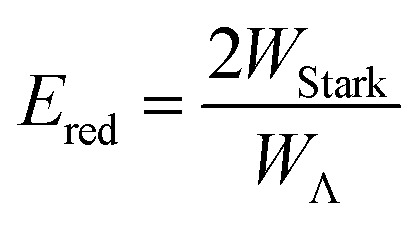
and4
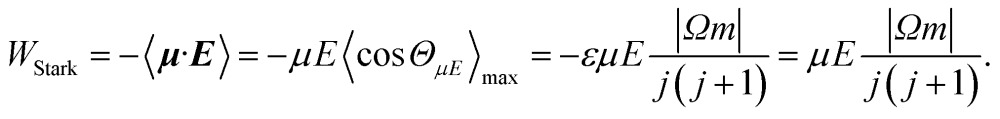



*W*_Λ_ is the Λ-doublet splitting between the *e* and *f* states, ***μ*** is the static dipole moment of NO(X) and ***E*** is the applied static electric field (taken as the LAB frame *z* axis), and *Θ*_*μE*_ is the angle between the two. The last of the above equations for *W*_Stark_ assumes that the *f* Λ-doublet state is selected through the hexapole.^26^

In the high field limit, *α* = *β* = 1 and if there is no applied field, *α* = 0 and 
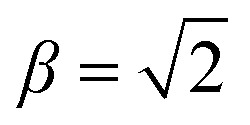
 such that the non-oriented *f* state wavefunction is recovered from eqn (1). At the field strength used in the experiments, *E* = 9.2 kV cm^–1^, the parameters *α* and *β* take the magnitudes 0.64 and 1.26, respectively. The values of *α* and *β* may be very slightly reduced at the time of interrogation, because the electric field in the interaction region is switched to velocity mapping potentials around 100 ns prior to the firing of the REMPI probe laser. However, in practice, we find little reduction in the integral steric asymmetry (see section D) up to delay times of around 200 ns, suggesting that the fraction of inelastic collisions occurring in the period between the switching of the voltages and the firing of the probe laser is relative small on the timescale of a few hundred nanoseconds.

**Fig. 3:**
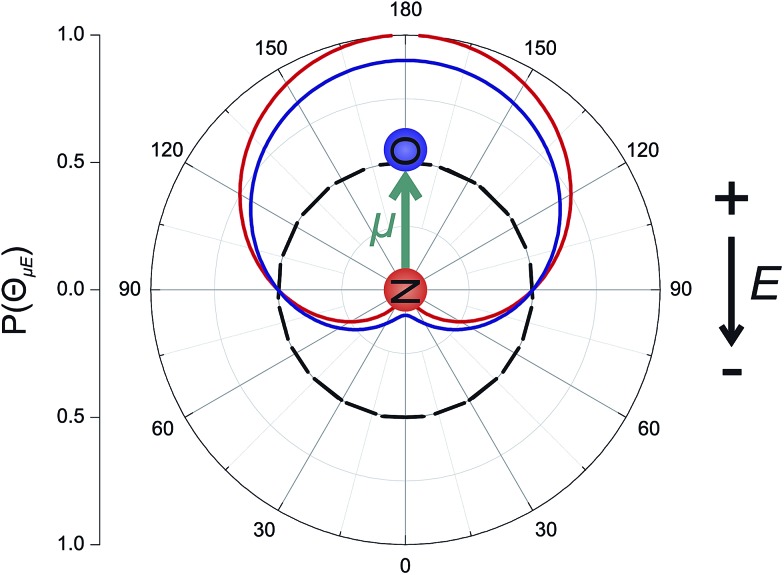
Polar plot of *P*(*Θ*_*μE*_), the probability distribution of the angle between the NO(X) bond axis vector and the electric field for an infinite field (red line), 9.2 kV cm^–1^ field (blue line) and no field (black dashed line). In this figure the dipole of NO is oriented towards the top of the page, and the electric field, *E*, points in the opposite direction, from top to bottom, such that the ‘N’ atom is directed towards the negative electrode, as indicated.

The orientation of the NO molecule in the field ***E*** depends on the relative signs of *α* and *β*. Fig. 3 shows a plot of the probability distribution of the angle between the dipole moment of the NO molecule and the electric field, given by5
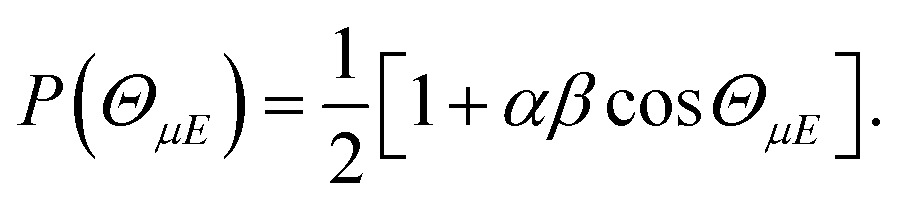



The figure shows the distribution for no applied field, the field used in the current experiments (*E* = 9.2 kV cm^–1^), and an infinite field. The permanent electric dipole of NO points from the negative N-atom to the positive O-atom. However, as the hexapole selects the low field seeking *f* Λ-doublet level, it is the ‘N’ end of the molecule that will be oriented towards the negative electrode (as shown in Fig. 3).^26^ This fact determines the relative signs of *α* and *β* in eqn (5): *α* and *β* must thus take opposite signs to ensure that the ‘N’ end of NO points towards the negative electrode in the static field (as shown in Fig. 2).

As illustrated in the Fig. 2, to a good approximation the field *E* can be aligned either parallel or anti-parallel to the initial relative velocity vector, defined as usual for inelastic scattering as ***k*** ≡ ***ν***_rel_ = ***ν***_Ar_ – ***ν***_NO_ (and similarly for the final relative velocity vector, ***k*′** ≡ *ν*′rel = *ν*′Ar – *ν*′NO). Thus when the ‘N’ end of the NO molecule is directed towards the velocity of the Ar in the centre-of-mass (CM) frame (labelled as ***w***_Ar_ in Fig. 2), then ***k*** will be parallel to the permanent electric dipole moment, ***μ***. Conversely, the opposite orientation, an ‘O’ end collision with Ar, can be obtained experimentally by reversing the direction of the applied field, in which case ***k*** will be anti-parallel to ***μ***.

### C Theoretical methods

In our theoretical simulations we assume, as indicated above, that the field ***E*** is aligned either parallel or anti-parallel to the initial relative velocity vector, ***k***.^24,25^

**Fig. 4:**
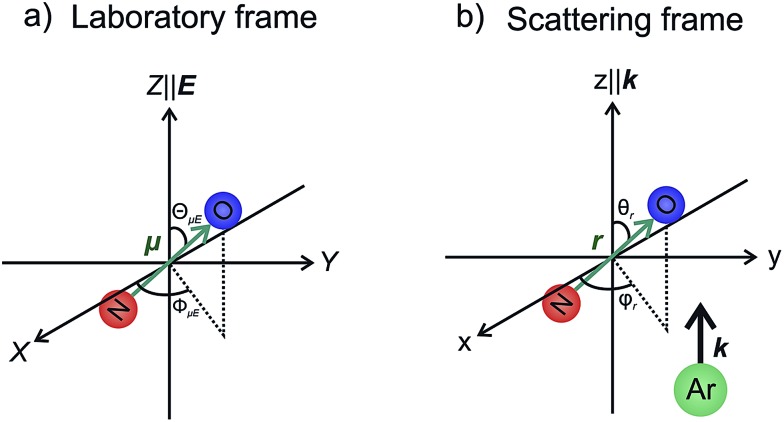
The laboratory (panel a) and scattering (panel b) frame coordinate systems used in the present work. See text for details.

The laboratory and scattering frames used in the present study are shown in Fig. 4. Laboratory frame is taken such that the *z*-axis lies in the direction of the electric field, whilst the scattering frame takes the relative velocity to be the *z*-axis, with the *xz* plane containing the initial and final relative velocities. In both the QM and QCT calculations we define ***r***∥***μ*** and ***R*** = ***R***_Ar_ – ***R***_NO_ (consistent with the definition of the initial relative velocity vector, ***k***, given above). The potential energy surface used in both sets of calculations^27^ is defined such that *γ* = 0 corresponds to the Ar–O–N configuration. Thus an ‘O’ end collision with Ar has ***r*** antiparallel to ***k***.

#### QM calculations

The scattering amplitude for oriented NO in an electric field, *E*, can be written as^24,25^
6
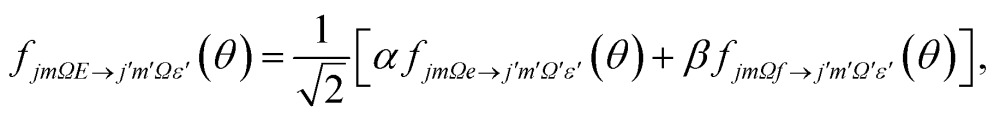
where *f*_*jmΩε*→*j*′*m*′*Ω*′*ε*′_(*θ*) is the scattering amplitude for the particular state-to-state transition at scattering angle *θ*. The corresponding oriented DCS can be obtained from the square modulus of the oriented scattering amplitude:7
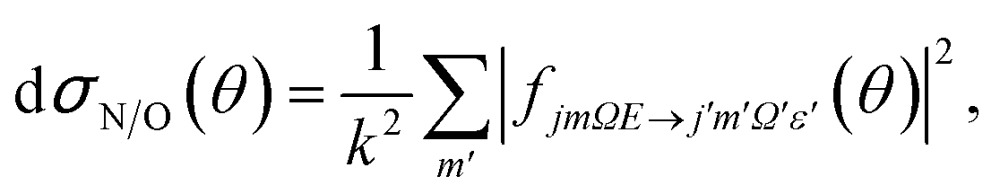
where d*σ*_N_(*θ*) and d*σ*_O_(*θ*) are the ‘N’-end and ‘O’-end DCSs, respectively. It follows from eqn (7) that the sum of the two oriented DCSs is given by the weighted (incoherent) sum of the two unoriented DCSs8
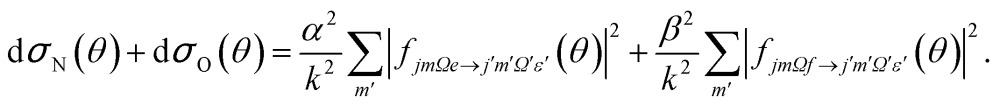



Similarly, the difference between the oriented DCSs will be given by9
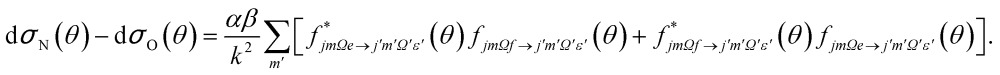



It can be seen from eqn (8) and (9) that in the case that the electric field is oriented along the relative velocity vector the difference depends on interference between scattering from the two initial Λ-doublet levels, whilst the sum does not. When investigating the differential steric asymmetry, it can be convenient to consider d*σ*_diff_(*θ*), the normalized difference DCS, which is defined as10
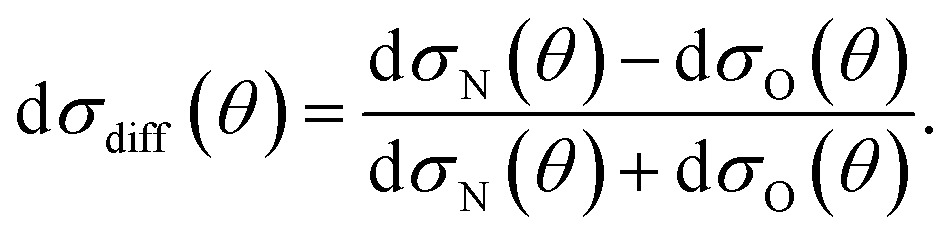



It should be noted that the integral steric asymmetry is not obtained by integrating the above expression for the differential steric asymmetry, but is determined by replacing the DCSs in the above expression by the corresponding NO orientation-specific integral cross sections.

The close-coupled quantum mechanical calculations presented in sections D and E are calculated using the HIBRIDON suite of codes.^28^ The calculations are run over a range of collision energies from of 500 cm^–1^ to 560 cm^–1^, and then averaged over the experimental collision energy distribution.^12^ The *V*_sum_ and *V*_diff_ NO(X) + Ar potential energy surfaces of Alexander^27^ are used in the calculations. The log derivative propagation method is used at short range (between 4.5 and 15 Bohr), with Airy propagation in the long range region (15 to 60 Bohr). A rotational basis including all states up to *j*′ = 20.5, and partial waves up to *J* = 160.5 are used in order to fully converge the DCSs.

#### Quasi-classical calculations

The bond axis distribution of the NO molecules is described by eqn (5). Using the method described in ref. 29–31, the oriented differential cross sections are calculated according to:11
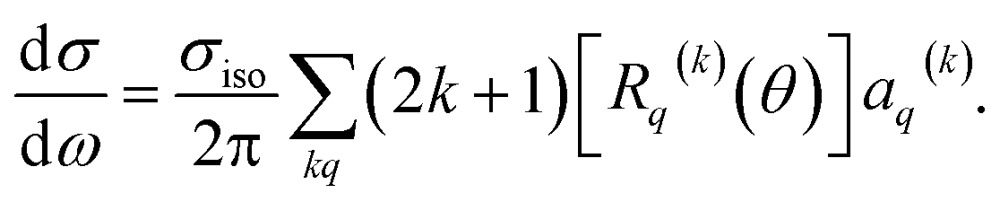



Here, *σ*_iso_ is the integral cross section for scattering with an isotropic initial bond axis distribution, [*R*_*q*_^(*k*)^(*θ*)] are the (real) intrinsic r-PDDCSs, calculated as detailed in ref. 30, and *a*_*q*_^(*k*)^ are the moments that describe the bond axis polarization in the scattering frame. Because it is assumed that the field is oriented either parallel or anti-parallel to the initial relative velocity, then 
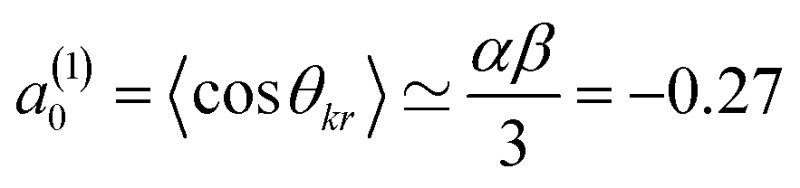
 for an ‘O’ end collision, and +0.27 for an ‘N’ end collision at the fields employed in the present experiments. *θ*_*kr*_ is the angle between the bond axis ***r*** and the relative velocity, ***k***. All other *a*_*q*_^(*k*)^ moments are zero for a *j* = 0.5 molecule, except for *a*(0)0 which is equal to unity.

The QCT calculations^32,33^ are run using only the *V*_sum_ PES of Alexander.^27^ At each collision energy we run 5 × 10^6^ trajectories. Since the NO bond length is fixed to its equilibrium value at all times the method of Lagrange multipliers are used to enforce the rigidity of the NO molecule. The final rotational quantum number, *j*, is determined by equating the square of the classical angular momentum, *j* ^2^, to *j* (*j* + 1)/*ħ* ^2^ and then rounded to the nearest integer. Note that the resulting *j* values are integer numbers. The maximum impact parameter, *b*_max_, at which trajectories are run is determined by monitoring the change in rotational quantum number, Δ*j* with increasing impact parameter. In this manner a value of *b*_max_ = 6.5 Å is chosen above which no trajectories with Δ*j* ≥ 0.5 are found.

As with the QM calculations, the QCT calculations are performed over a grid of collision energies from 500 cm^–1^ to 560 cm^–1^ with a spacing of 15 cm^–1^, and the theoretical data are averaged over a Gaussian collision energy distribution with a mean of 530 cm^–1^, and a full-width-at-half-maximum (FWHM) of 50 cm^–1^.^12^

## Results and discussion

### A Integral steric asymmetry

Consistent with the preceding discussion, the dimensionless integral steric asymmetry, *S*, is defined as^18,19,24,25^
12
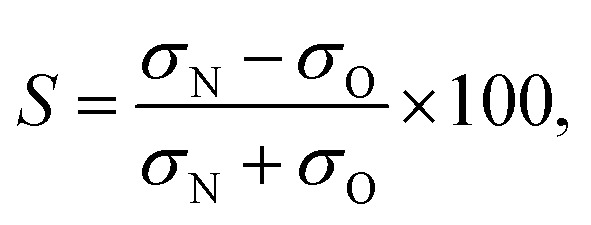
where *σ*_N_ and *σ*_O_ are the oriented integral cross sections, which are obtained by integrating the oriented differential cross sections over all scattering angles. The steric asymmetry can be obtained from the experimental images by integrating the intensity of the ion images corresponding to the two orientations and then taking the normalized difference. It is not possible to directly measure the integral cross section from the ion images, however the calibration factor to convert signal intensity to absolute cross section will be very similar for the two orientations, so it will cancel when the normalized difference is calculated according to eqn (12).

**Fig. 5:**
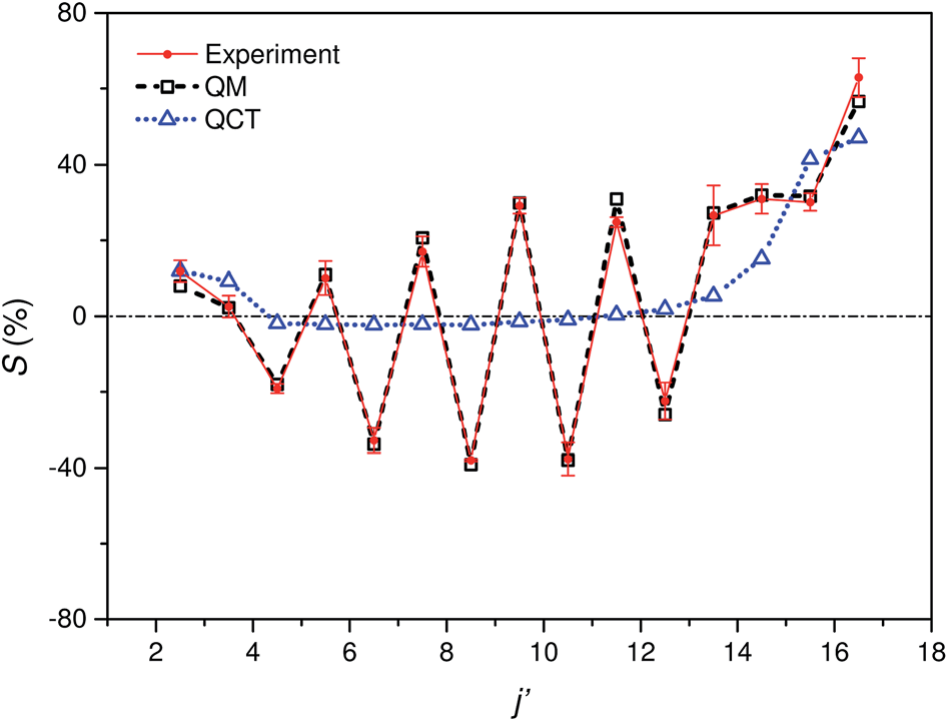
Comparison of experimental (red continuous line with points and error bars) and quantum mechanical (black dashed line with open squares) integral steric asymmetry, as defined in eqn (12) of the main text, for transitions leading to the *e* final Λ-doublet levels. Note that a positive value of *S* indicated a preference for N-end collisions. The corresponding QCT data are shown as a blue dotted line with triangles, as indicated.

Fig. 5 shows the experimental integral steric asymmetry for transitions ending in *e* Λ-doublet states compared to the quantum mechanical calculations. The agreement between the experimental steric asymmetry and quantum mechanical calculations is very good for all *j*′. The steric asymmetry is large and shows an alternation in sign with *j*′ state, as has been seen in previous theoretical^25^ and experimental^19^ work. It should be noted that the integral steric asymmetry is insensitive to the choice of final Λ-doublet level.^3,25^ There has previously been some discussion as to the correct sign of the steric asymmetry.^26,34^ In the current experiments we find that the measured steric asymmetry has the same sign as the quantum mechanical calculations, for which *S* is large and positive at high *j*′, indicating an ‘N’ end preference to populate high rotational states (see further below).

The QCT calculations are unable to reproduce the oscillations found with Δ*j* and for most of the final states the predicted integral steric asymmetry is nearly zero. We conclude that the oscillatory behaviour of the steric asymmetry with Δ*j* is a purely QM effect, a fuller discussion of which has been given previously in ref. 3 and 25. However, as we have seen, at high Δ*j* the QCT calculations predict a preference for ‘N’ end collisions, as also observed experimentally and in the QM calculations. This preference is as expected on the basis of a simple classical ball and stick model: Because the CM of NO is slightly displaced towards the O-atom, collisions with the ‘N’ end can apply more torque, and therefore lead to greater rotational excitation.

The excellent agreement between experiment and QM theory presented here provides confidence in both the experimental and QM theoretical treatments employed in the study of differential steric asymmetry.

### B Differential steric asymmerty

**Fig. 6:**
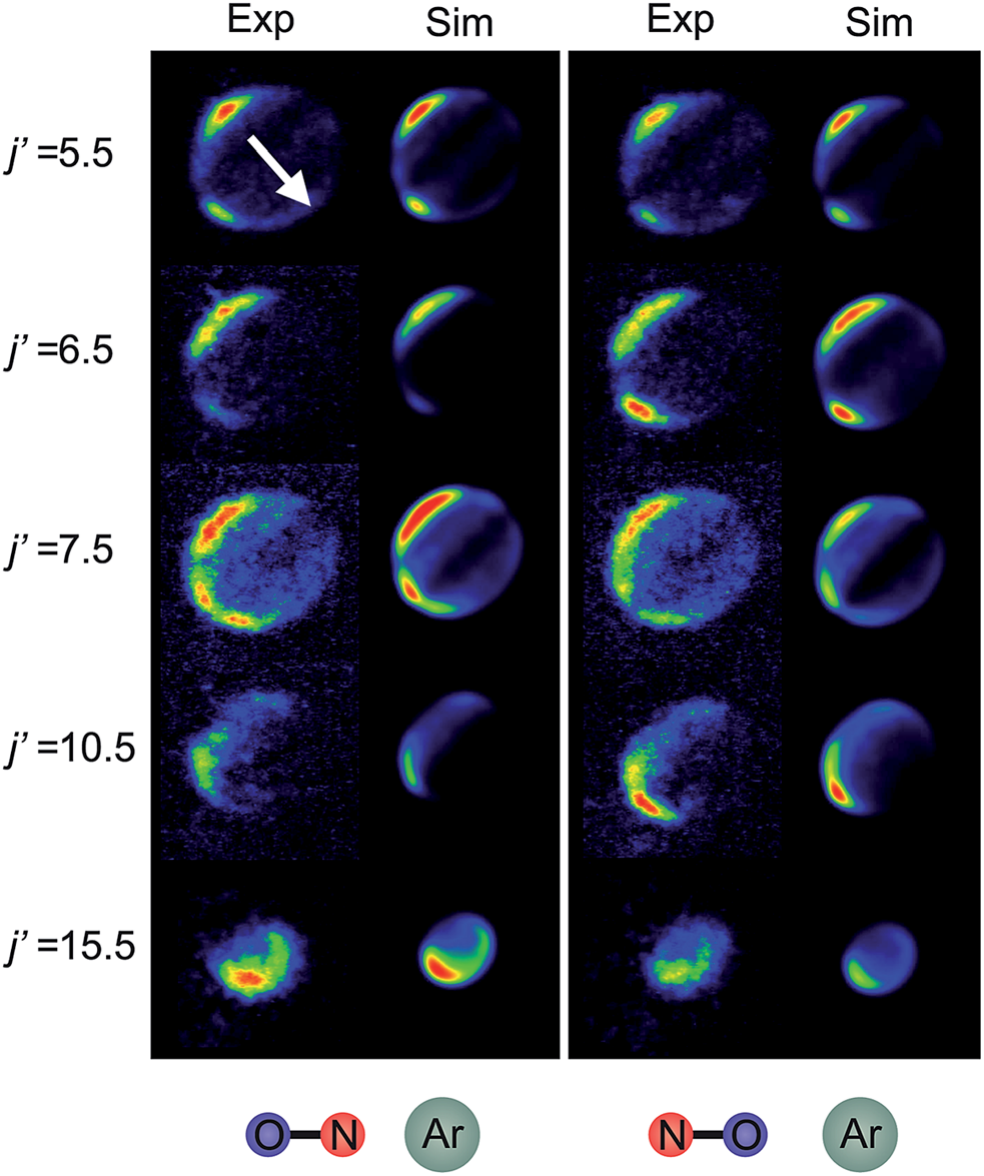
Experimental (1^st^ and 3^rd^ columns) and simulated (2^nd^ and 4^th^ columns) ion images for a selection of spin orbit conserving transitions into the *e* Λ-doublet level. The left hand panel shows images for collisions where the ‘N’ end of the molecule is preferentially oriented towards the Ar, whilst the right hand panel shows images for the ‘O’ end collisions. The white arrow in the top left panel shows the direction of the relative velocity (as defined in section B).

Fig. 6 shows experimental and simulated ion images for transitions into *j*′ = 5.5*e*, 6.5*e*, 7.5*e*, 10.5*e*, and 15.5*e* for collisions with either the ‘N’ (left columns) or ‘O’ end (right columns) of NO. For all these transitions the NO molecule remains in its lowest spin–orbit and vibrational states. In the simulation we use a Monte-Carlo method^12^ to generate a set of basis images and then use a sum of these weighted appropriately according to the DCS predicted by the quantum scattering calculations. The images are slightly distorted from circularity in the direction perpendicular to the relative velocity. This is a consequence of deformities in the velocity mapping field due to the presence of the rods, an effect which we have modelled and incorporated into the simulation and fitting procedures.

The white arrow in the top left of Fig. 6 indicates the direction of the relative velocity, *k* (defined in section B). The asymmetry about *k* in both the experimental and simulated images is due to the difference in the lab-frame velocities of the scattered NO molecules in different areas of the image.^12^ The extent of both the experimental and simulated images decreases with increasing *j*′, as more of the collision energy is transferred to rotational motion, resulting in a smaller outgoing velocity of the NO. For all states (except for *j*′ = 5.5) differences in intensity between the ‘N’ and ‘O’ orientations are apparent, in both the experimental and simulated images.

**Fig. 7:**
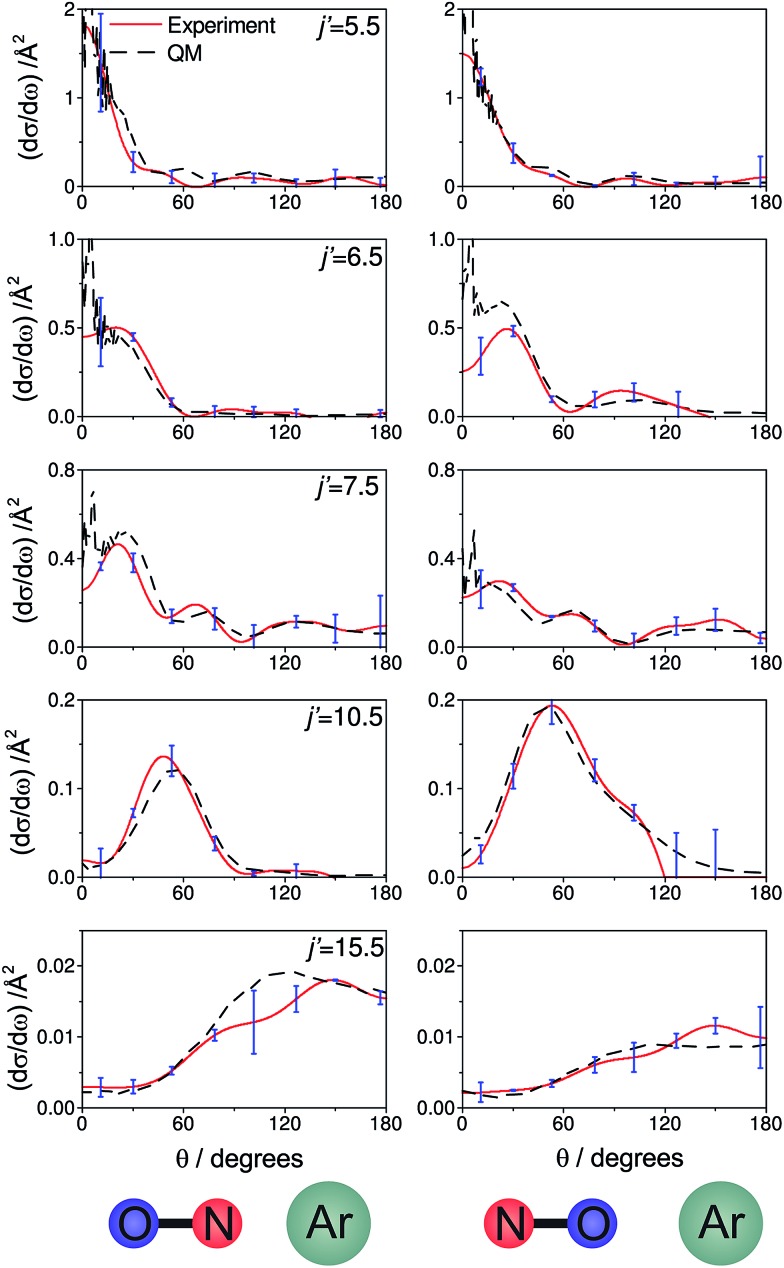
The DCSs determined experimentally from the images shown in Fig. 6 (red continuous lines) and the corresponding QM DCSs (black dashed lines). The data shown are for a selection of spin orbit conserving transitions into the *e* Λ-doublet level. The error bars associated with the experimental data (shown in blue) represent 95% confidence limits.

Fig. 7 shows a comparison of the experimentally determined oriented DCSs [derived by means of the method outlined in the ESI† and in ref. 12] and the DCSs predicted by our quantum scattering calculations. The agreement is generally very good, even down to the subtle differences in the oriented DCSs. See, for example, the small peak at around 100° in the *j*′ = 6.5 NO–Ar data which is all but absent for the ON–Ar orientation. The orientation of the NO bond axis prior to the collision has a significant effect on the relative intensities of the peaks in the angular distributions, but not on their number or position. The QM calculations reproduce very well the relative magnitudes of the peaks seen in the experimental DCS (for example, *j*′ = 10.5), as would be expected given the excellent agreement seen for the integral steric asymmetries in section D.

For both bond orientations, the main peak in the DCS shifts from forward scattering (the direction of the motion of the NO is little altered by the collision) at low *j*′ (5.5 ≤ *j*′ ≤ 7.5) to sideways scattering for middle *j*′ (*j*′ = 10.5) and then to backward scattering for the highest state (*j*′ = 15.5).^35^ This is to be expected: transitions with small changes in *j* result primarily from high impact parameter ‘glancing’ trajectories, whereas a large degree of rotational excitations necessitates low impact parameter ‘head-on’ collisions. For *j*′ = 5.5, the images and the DCSs show little dependence on the orientation of the NO bond axis.

**Fig. 8:**
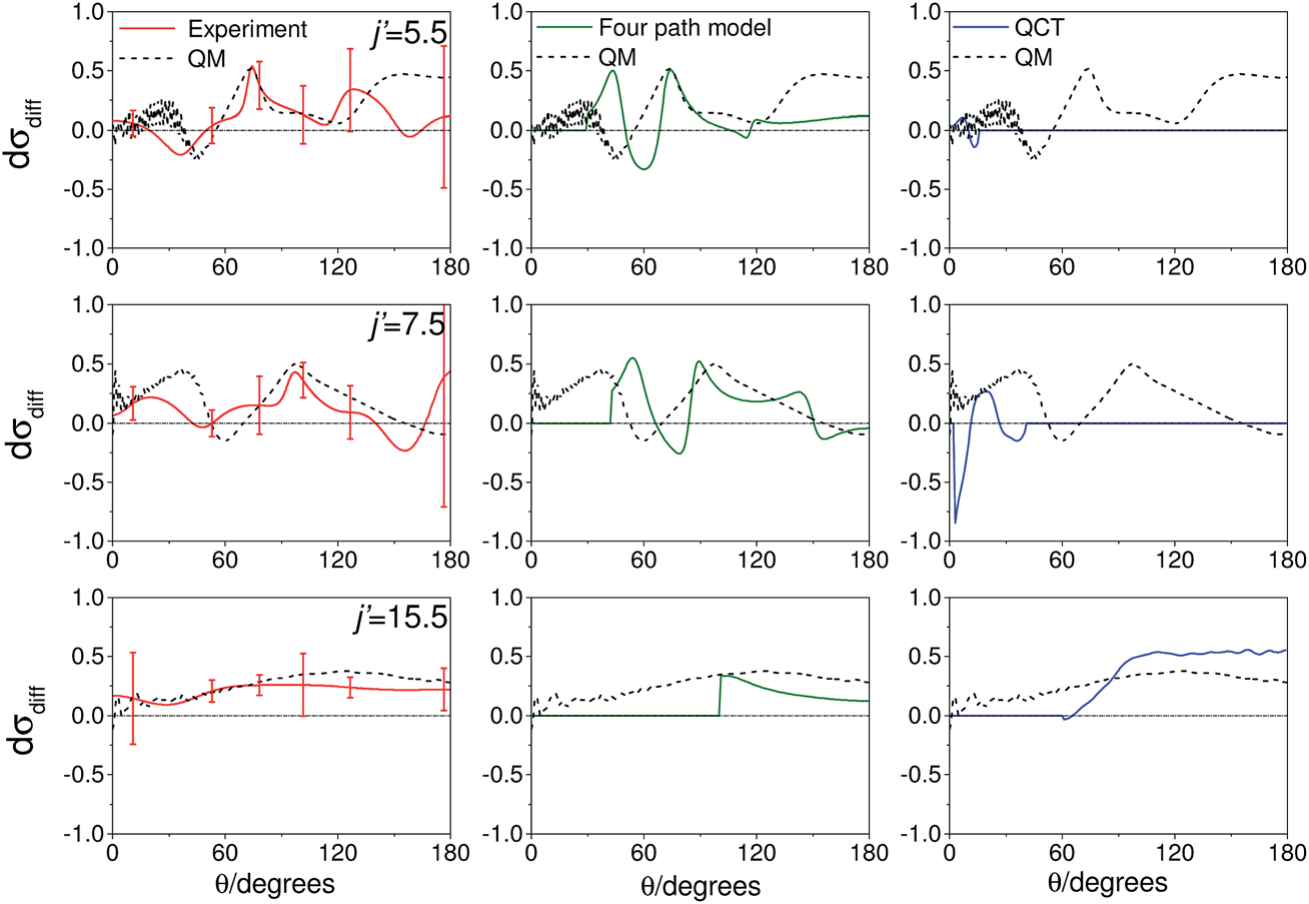
Normalized difference DCSs, d*σ*_diff_(*θ*), as defined in eqn (10), for transitions to *j*′ = 5.5, 7.5, 15.5*e*. In all columns the quantum scattering predictions are shown by the black line. The experimental d*σ*_diff_(*θ*) (red line) is shown in the left column, four-path model calculation (green line) in the middle column and QCT calculation (blue line) in the right column. The error bars associated with the experimental data represent 95% confidence limits. Note that a positive value of d*σ*_diff_(*θ*) indicated a preference for N-end collisions.

As noted in section C, it can be instructive to consider the normalized difference DCSs, as defined in eqn (10). The experimental and quantum mechanical d*σ*_diff_(*θ*)'s for a low, middle and high *j*′ state are shown in the left hand column of Fig. 8. A positive value indicates more inelastic ‘N’ end collisions and a negative value, more inelastic ‘O’ end collisions.

Here, too, the agreement between experiment and QM theory is reasonable, despite the fact that the errors in the individual oriented DCSs will be amplified through propagation when calculating the normalized difference. It can be seen that the experimental normalized difference DCSs oscillate between approximately –0.2 and 0.5 for *j*′ = 5.5 and 7.5 (compared with limiting values of +1 and –1). For these states, the range of d*σ*_diff_(*θ*) over which the normalized difference DCSs oscillate is well predicted by the QM calculations, as are the frequency and positions of the oscillations for *θ* ≤ 100°. The largest discrepancies occur in the backwards direction for *j*′ = 5.5 and 7.5, where the experimental scattered intensity is low, and therefore the experimental errors in the normalized difference DCSs are large.

For *j*′ = 15.5, no such oscillations are observed in the experimental or QM data, so that d*σ*_diff_(*θ*) is positive over almost the entire angular range. As with the integral steric asymmetry, the simple ball and stick model mentioned in the preceding section goes some way to explain this preference for ‘N’ end collisions at high Δ*j*.

QCT calculations (shown in the right hand column) predict DCSs which are nearly independent of the initial orientation of the NO molecule. They fail to reproduce the structure observed in the quantum calculations. Other than at high *j*′, for which the steric preference can be explained by the simple ball and stick model, the general failure of a classical picture confirms that the steric asymmetry in the angular distributions is due to constructive or destructive quantum interference between trajectories which scatter off the two different ends of the NO molecule.

A simple qualitative (but far from quantitative) explanation is provided by a ‘four-path’ model, which treats the collision as that of a hard sphere and a hard ellipsoid, restricted to four limiting paths.^12^ It has previously been used to predict the position of the parity dependent oscillations observed in the DCSs of the NO(X) + Ar system.^12,36^ Within this model, the angular dependence of the oriented inelastic cross sections reflects interference between relative phase shifts associated with scattering off different parts of the molecule. The expression for the normalized difference DCSs in the four path model is given by (for details see ESI†)13
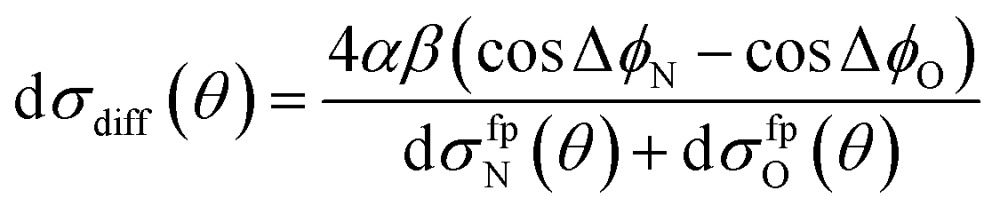
with

14 d*σ*fpN(*θ*) + d*σ*fpO(*θ*) = *α*^2^[6 + 4(cos Δ*φ*_N_ + cos Δ*φ*_O_) + 2 cos(Δ*φ*_N_ – Δ*φ*_O_)] + 2*β*^2^[1 – cos(Δ*φ*_N_ – Δ*φ*_O_)].

Here Δ*φ*_N_ and Δ*φ*_O_ are the relative phase shifts associated with scattering off either of the two ends and the side of the NO(X) molecules and are defined in the ESI.†Eqn (13) indicates that the steric asymmetry arises from a quantum interference between scattering from the two ends of the NO(X) molecule, in agreement with the conclusions of the quasi-quantum treatment presented in ref. 3.

Note that the four path model only provides information about the variation of the oriented differential cross section with scattering angle, but it cannot predict the absolute magnitude, as it neglects the geometric cross section,^36^ which contains information on the relative weights of each path. Therefore, in order to calculate the normalized difference oriented differential cross section, the individual four path model oriented differential cross sections are weighted by the quantum mechanical integral cross sections.

The middle column of Fig. 8 shows that the four-path model does predict oscillations whose modulation depths and ‘wavelengths’ correspond roughly to the predictions from quantum scattering calculations. Unfortunately, the four-path model is unable to describe weak, non hard-shell, collisions and hence cannot be applied to small-angle (large impact parameter) scattering.

The decrease in the number of oscillations in d*σ*_diff_(*θ*) with increasing rotational excitation can be rationalized in terms of the outgoing de Broglie wavelength of the system. As Δ*j* increases, the relative NO–Ar velocity after collision decreases and hence the de Broglie wavelength increases.

## Conclusions

In a study of collisions of NO with Ar, we have used a static electric field in the interaction region to orient the NO bond axis such that either the ‘N’ or ‘O’ end is directed towards the incoming Ar atom. Fast switching of the orientation electrodes allowed us to employ velocity map ion imaging to determine the differential cross section for the oriented scattering, providing information on the three vector ***k***–***r***–***k*′** correlation. These fully quantum state-resolved stereodynamical experiments allow for the study of the NO + Ar system in unprecedented detail. Oriented differential cross sections for a selection of final rotational states have been presented and agreement with quantum mechanical calculations has been found to be very good.

Calculation of the normalised difference DCS using QM, QCT and semi-classical models has revealed that the differential steric asymmetry tells us how the interference from scattering from the two ends of the molecule varies with scattering angle. The method described in this paper for orientation of the NO(X) bond axis could be applied to other open shell diatomic molecules for investigating the stereodynamics of different systems. Further experimental study of the collisions of oriented NO with other diatomic molecules would also provide additional insight into the subtle stereodynamics of inelastic scattering. Of particular interest might be collisions of NO with HD or OH, recently studied under crossed-beam conditions.^37^

By combining the current experiment with linearly or circularly polarized laser light, it would also be possible to determine the alignment or orientation of the rotational angular momentum, *j*′.^6,8,11,15^ Measurement of this “full” four vector correlation^38^ between the bond vector and relative momenta (or, equivalently, between the rotational and relative momenta) of the scattering partners before and after the scattering event would provide maximal information on the underlying intermolecular forces, free of incoherent averaging over multiple quantum states and directions.^16,39,40^
