# Supplementary material for: Steric effects and quantum interference in the inelastic scattering of NO(X) + Ar
Source: Chem Sci. 2015 Feb 3;6(4):2202–10. doi: 10.1039/c4sc03842h (PMC5485563; doi:10.1039/c4sc03842h)
Supplement: Supplementary file 1 [file SC-006-C4SC03842H-s001.pdf]

Electronic Supplementary Material (ESI) for Chemical Science.
This journal is © The Royal Society of Chemistry 2015

Supplementary Information 17 January 2015

'Steric effects and quantum interference in the inelastic scattering of NO(X) + Ar'

Nichols *et al.*

Here we discuss the following topics: I) the method used to analyse the experimental images; II) the four path model

1 Image analysis

The method of data analysis for the differential cross section images has been discussed in detail previously[1,2], so only the relevant details will be given here. The probability of detecting a single scattering event, n, is given by[1]:

$$I(x_n, y_n) = \mathscr{A}(x_n, y_n) P_{\text{scatt}}(\theta) P_{\text{V/H}}(\theta_n; \chi_p^n), \quad (1)$$

where $\mathscr{A}(x_n, y_n)$ is the apparatus function for a pixel of position (x_n, y_n) on the detector, $P_{\text{scatt}}(\theta)$ is the angular scattering distribution (proportional to the DCS) and $P_{\text{V/H}}$ is the polarisation dependent transition probability. The Euler angles, χ_p, can be determined from the relative orientation of the scattering frame (defined by $\boldsymbol{k}$ and $\boldsymbol{k}'$) and the laser frame (defined by the electric vector of the light, and the direction of laser propagation)[1]. The polarisation dependent transition probability can be expressed in terms of the Hertel-Stoll renormalized PDDCSs:

$$P_{\text{V/H}}(\theta_n; \chi_p^n) = 1 + \sum_{kq} \rho_{q\pm}^{\{k\}}(\theta_n) F_{q\pm}^{\{k\}}(\chi_p^n). \quad (2)$$

The $F_{q\pm}^{\{k\}}(\chi_p^n)$ functions are defined in Ref.[1] and contain the geometric information needed to calculate the contribution of a particular PDDCS. Note that, when substituted into Eq. (1), the first term in Eq. (2) corresponds to the probability of detecting a single scattering event without polarisation, and is that which is required to determine the DCS[1].

Obtaining the differential cross sections

When fitting the experimental images to obtain the DCSs, the QM $\rho_{q\pm}^{\{k\}}(\theta)$ renormalized PDDCSs are assumed to be correct, and are used to account for the polarization of $\boldsymbol{j}'$ following the collision. The DCS can then be expanded in terms of Legendre polynomials according to:

$$P_{\text{scatt}}(\theta) = \sum_{l} \frac{2l+1}{2} a^{(l)} P_l(\cos\theta). \tag{3}$$

where $a^{(l)}$ are the expansion coefficients and $P_l(\cos\theta)$ are the lth Legendre polynomial. The intensity of the experimental image can then be fit as:

$$I_{\text{exp}}(x,y,\chi_p) = \frac{1}{2}\sum_{l}(2l+1)a^{(l)}B_l(x,y,\chi_p), \tag{4}$$

where the basis functions are given by:

$$B_l(x,y,\chi_p) = P_l(\cos\theta)\mathscr{A}(x,y)\left[1+\sum_{kq}\rho_{q\pm}^{\{k\}}(\theta_n)F_{q\pm}^{\{k\}}(\chi_p)\right]. \tag{5}$$

The experimental images recorded using horizontally and vertically polarized light were fitted separately using up to 12 basis functions and the two fits averaged to obtain the experimental DCSs presented in the main paper.

2 Four path model

A simple hard shell ellipse model, which approximates the scattering process as occurring via four limiting pathways can be adapted to predict the stereodynamics of oriented NO + Ar scattering. It has previously been used to predict the position of the parity dependent oscillations observed in the DCSs of the NO(X) + Ar system[3,4]. The scattering amplitude can be written[4]

$$f_{f\leftarrow i}(\theta) \propto 1 + \mathrm{e}^{-i(\Delta\phi_{\mathrm{N}}-\pi\Delta p/2)} + \mathrm{e}^{+i\pi\Delta p/2} + \mathrm{e}^{-i\Delta\phi_{\mathrm{O}}}, \tag{6}$$

where $\Delta p = p' - p$ is the change in the total NO(X) parity, with $\Delta p/2 = 0$ in the case of parity conserving transitions and $\Delta p/2 = 1$ for parity changing transitions. $\Delta\phi_{\mathrm{O}}$ and $\Delta\phi_{\mathrm{N}}$ are the relative phase shifts associated with scattering from either the pointed 'N' or 'O' ends of the molecule and the flatter middle[4]. For a near homonuclear molecule these phase shifts can be written[4–6]

$$\Delta\phi_n = (\Delta j_{\theta,n}^2 - j'^2)^{1/2} - j' \cos^{-1}\left(\frac{j'}{\Delta j_{\theta,n}}\right), \tag{7}$$

where $j_{\theta,n}$ is the amount of momentum transferred if the NO(X) molecule is struck at the $n = \mathrm{N}$ or $n = \mathrm{O}$ end of the molecule and scattered though an angle θ,

$$\Delta j_{\theta,n} = \Delta j_{\mathrm{max},n} \sin\left(\frac{\theta}{2}\right), \tag{8}$$

and $\Delta j_{\mathrm{max,n}}$ is the maximum amount of momentum transferred if the molecule is perfectly backscattered. Assuming a hard shell potential where A_n is the major semi-axis for end n of the ellipsoid and B_n is the minor semi-axis

$$\Delta j_{\mathrm{max},n} = 2k(A_n - B), \tag{9}$$

where $k = p/\hbar$ is the wavevector, and p is the linear momentum.

It follows from Eq. (6) that the parity conserving and changing scattering amplitudes can be written[4]

$$\begin{aligned} f_{\mathrm{cons}}(\theta) &\propto 2 + \mathrm{e}^{-i\Delta\phi_{\mathrm{N}}} + \mathrm{e}^{-i\Delta\phi_{\mathrm{O}}} \\ f_{\mathrm{chang}}(\theta) &\propto \mathrm{e}^{-i(\Delta\phi_{\mathrm{N}} - \pi)} + \mathrm{e}^{-i\Delta\phi_{\mathrm{O}}}\,. \end{aligned} \tag{10}$$

These expressions can be used in equation (7) of the main paper to obtain the expres-

sions for the four path model differential cross sections

$$\begin{aligned} d\sigma_{N}^{fp}(\theta) &\propto \frac{1}{2}\left[\alpha^2\left\{6+4\left(\cos\Delta\phi_N+\cos\Delta\phi_O\right)+2\cos\left(\Delta\phi_N-\Delta\phi_O\right)\right\}\right. \\ &+ \left. 2\beta^2\left(1-\cos(\Delta\phi_N-\Delta\phi_O)\right)-4\alpha\beta\left(\cos\Delta\phi_O-\cos\Delta\phi_N\right)\right] \qquad (11) \\ d\sigma_{O}^{fp}(\theta) &\propto \frac{1}{2}\left[\alpha^2\left\{6+4\left(\cos\Delta\phi_N+\cos\Delta\phi_O\right)+2\cos\left(\Delta\phi_N-\Delta\phi_O\right)\right\}\right. \\ &+ \left. 2\beta^2\left(1-\cos(\Delta\phi_N-\Delta\phi_O)\right)+4\alpha\beta\left(\cos\Delta\phi_O-\cos\Delta\phi_N\right)\right]. \qquad (12) \end{aligned}$$

As given in the main text, the expression for the normalized difference DCSs is therefore given by:

$$d\sigma_{\mathrm{diff}}(\theta)=\frac{4\alpha\beta\left(\cos\Delta\phi_N-\cos\Delta\phi_O\right)}{d\sigma_{N}^{fp}(\theta)+d\sigma_{O}^{fp}(\theta)} \qquad (13)$$

with

$$\begin{aligned} d\sigma_{N}^{fp}(\theta)+d\sigma_{O}^{fp}(\theta) &= \alpha^2\left[6+4\left(\cos\Delta\phi_N+\cos\Delta\phi_O\right)+2\cos\left(\Delta\phi_N-\Delta\phi_O\right)\right] \\ &+ 2\beta^2\left[1-\cos\left(\Delta\phi_N-\Delta\phi_O\right)\right]. \qquad (14) \end{aligned}$$

References

[1] M. Brouard, H. Chadwick, C. J. Eyles, B. Hornung, B. Nichols, F. J. Aoiz, P. G. Jambrina and S. Stolte, *J. Chem. Phys.*, 2013, **138**, 104310.

[2] M. Brouard, H. Chadwick, C. J. Eyles, B. Hornung, B. Nichols, J. M. Scott, F. J. Aoiz, J. Kłos, S. Stolte and X. Zhang, *Mol. Phys.*, 2013, **111**, 1759.

[3] C. J. Eyles, M. Brouard, C. H. Yang, J. Kłos, F. J. Aoiz, A. Gijsbertsen, A. E. Wiskerke and S. Stolte, *Nat. Chem.*, 2011, **3**, 597–602.

[4] C. J. Eyles, M. Brouard, H. Chadwick, F. J. Aoiz, J. Kłos, A. Gijsbertsen, X. Zhang and S. Stolte, *Phys. Chem. Chem. Phys.*, 2012, **14**, 5420–5439.

[5] H. J. Korsch and R. Schinke, *J. Chem. Phys.*, 1980, **73**, 1222.

[6] H. J. Korsch and R. Schinke, *J. Chem. Phys.*, 1981, **75**, 3850.
